# Prescriptions

**Published:** 1896-11

**Authors:** 


BRONCHITIS.

℞. Liq. ammonii acetat....... ℥ss.

Syr. ipecac............... ʒj.

Liq. morph. sulph. (U. S. P.) ♏xl.

Syr. acaciæ.............. ℥j.

Aquæ.................... ℥iss.

M. Sig.: A teaspoonful every two hours for a child two years old. (*In capillary bronchitis.*)—*Meigs and Pepper.*

BUNIONS.

℞. Tinct. iodinii............
Tinct. belladonnæ.....aa ʒij.
M. Sig.: Apply twice daily with a brush.—*Annual Univ. Med. Sci.*

HÆMATURIA.

℞. Tinct. ferri muriat ....♏xxx.

Tinct. digitalis ........♏xv.

Aqæ menthæ pip....... ℥iss.

M. Sig.: To be repeated every four hours.—*Aitken.*

HEMORRHOIDS.

℞. Acidi carbolici.... ...... ʒij.

Acidi tannici .............. ʒj.

Alcoholis.................. ʒiv.

Glycerinæ... .............. ℥j.

M. Sig.: Hypodermic injection for piles.—*Girard.*

ASTHMA.

℞. Potassii iodidi...........
Chloral hydrat ..........aa ʒj.
Syr. aurantii cort ....... ℥j.
Aq. aurantii flor........ad ℥ij.
M. Sig.: A tablespoonful once or twice during the attack.—*Lazarus, Annual Univ. Med. Sci.*

